# Epidemiological and risk analysis of the H7N9 subtype influenza outbreak in China at its early stage

**DOI:** 10.1007/s11434-013-5880-5

**Published:** 2013-04-27

**Authors:** QingYe Zhuang, SuChun Wang, MeiLi Wu, Shuo Liu, WenMing Jiang, GuangYu Hou, JinPing Li, KaiCheng Wang, JianMin Yu, JiMing Chen, JiWang Chen

**Affiliations:** 1 China National Avian Influenza Professional Laboratory, China Animal Health and Epidemiology Center, Qingdao, 266032 China; 2 College of Animal Science and Veterirary Medcine, Qingdao Agricultural University, Qingdao, 266032 China; 3 The Institute for Personalized Respiratory Medicine, University of Illinois at Chicago, Chicago, IL 60612 USA

**Keywords:** H7N9, avian influenza, virus, outbreak, epidemiology, risk

## Abstract

Dozens of human cases infected with H7N9 subtype avian influenza virus (AIV) have been confirmed in China since March, 2013. Distribution data of sexes, ages, professions and regions of the cases were analyzed in this report. The results showed that the elderly cases, especially the male elderly, were significantly more than expected, which is different from human cases of H5N1 avian influenza and human cases of the pandemic H1N1 influenza. The outbreak was rated as a Grade III (severe) outbreak, and it would evolve into a Grade IV (very severe) outbreak soon, using a method reported previously. The H7N9 AIV will probably circulate in humans, birds and pigs for years. Moreover, with the driving force of natural selection, the virus will probably evolve into highly pathogenic AIV in birds, and into a deadly pandemic influenza virus in humans. Therefore, the H7N9 outbreak has been assumed severe, and it is likely to become very or extremely severe in the future, highlighting the emergent need of forceful scientific measures to eliminate any infected animal flocks. We also described two possible mild scenarios of the future evolution of the outbreak.

Human cases infected with H7N9 subtype avian influenza virus (AIV) emerged in China in recent months [1–3]. The H7N9 influenza toll has climbed to 91 on April 19. Among the 91 confirmed cases, 17 died. This outbreak aroused great global concerns, due to it being the first time in recent decades that an AIV has caused multiple human severe infections in a wide region during a short period. It is highly desired to timely reveal the epidemiological features and risk of the outbreak [1–3]. We elucidated here the sex, age, professional, and geographical distribution of the first 91 cases, the transmission and severity of the outbreak, as well as the risk of the outbreak in the future.

†These authors contributed equally to this work.
*Corresponding author (email: jmchen66@yahoo.cn)

## 1 Data sources and analysis methods

Case data were from online official reports. Differences with $P$ <0.05 using the $\chi^2$ test were assumed to be statistically significant, if not specified.

## 2 Clinical and demographic characteristics of the first 91 cases

Of the first 91 cases, 86 were of known clinical signs. Among them, 88.37% (76/86) developed severe and acute respiratory symptoms including pneumonia, and 17 had died. The case fatality rate was approximately 18.68%, significantly higher than that of the SARS outbreak in China in 2003 (6.44%) [4]. From the data of 13 fatalities of known relevant data, the interval between the occurrence of symptoms and death of the fatal cases was 12.22 (mean) ± 6.76 d (standard deviation).

Of the first 91 cases, 87 were of known sexes and ages. Among them, males ($n$ = 61) were significantly more than females ($n$ = 26), and 3 were 0–19-year-old, 13 were 20–39-year-old, 23 were 40–59-year-old, 48 were ⩾60-year-old. Compared with the age distribution of the total population in China [5], the cases were significantly fewer for the groups of 0–19-year-old and 20–39-year-old, and significantly more for the group of ⩾60-year-old (Figure 1).

The sex and age distribution of the H7N9 cases was quite different from that of the human cases of H5N1 avian influenza occurring in 2003–2006, and that of human cases of the pandemic H1N1 influenza in 2009 (Figure 1). Both of the later two outbreaks exhibited no sex difference. Moreover, for the H5N1 and pandemic H1N1 cases, elder cases of >60-year-old were significantly fewer than expected (both covering <5% of the total cases), and young cases of <40-year-old, especially for those of 0–19-year-old, were significantly higher than expected, both covering >80% of the total cases [6,7]. Figure 1 also showed that the age distribution of H7N9 cases was similar to the SARS outbreak in 2003 in China in that the group of 0–19-year-old were significantly fewer than expected, but different from the SARS outbreak in that elder cases of >60-year-old were significantly fewer than expected for the SARS outbreak [4].

Among the 87 cases with known ages, males of ⩾60-year-old ($n$ = 36) were significantly more than females of ⩾60-year-old ($n$ = 12), but males of <60-year-old ($n$ = 25) were not significantly more than females of <60-year-old ($n$ = 14).

The elderly are usually weaker than the young, and elder males are usually further weaker than elder females, which could be seen from the fact that males are of the mean lifetime 5 years shorter than that of females in China [5]. Possibly due to the virus having not been fully adapted to humans, it invaded mainly the weak. Therefore, especially elder male cases were significantly more than expected.

Most (86) of the first 91 cases were from the lower reaches of the Yangtze River covering three provinces (Anhui, Jiangsu and Zhejiang) and the city of Shanghai, and five cases were from Henan province and Beijing. Their infection sites formed an area larger than 500000 $km^2$.

Of the first 91 cases, 71 were of known professions. Among them, 95.77% (68/71) were the retired, peasants, bird transporters or sellers, cookers, or of no formal professions, with only three exceptions in the groups of officials, students, teachers, medical staffs, etc. More than a half of the cases were of recent history exposed to live birds, especially live birds in markets. Of the first 91 cases, only two pairs were from the same families, and no sound evidences supported human-to-human transmission between them. Among the total of 1108 close contacts of the first cases reported online, only two (namely the two in the two pairs from the same families) were identified as H7N9 cases. All these data suggest that the virus could not transmit among humans. This further suggests that most of the first 91 cases were infected with the virus from infected animals.

## 3 Severity rating of the H7N9 influenza outbreak

Severity rating of an outbreak of infectious diseases is important in infection control. Currently, a method has been formulated to rate the severity of a human infectious disease outbreak [8]. This method involves scoring four aspects: the

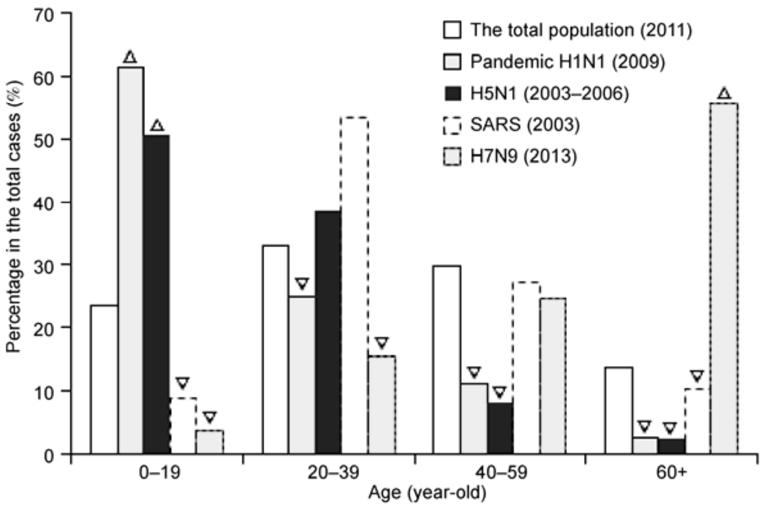

**Figure 1** The age distribution of the total population in 2011, human cases of pandemic H1N1 influenza in 2009, human cases of H5N1 avian influenza in 2003–2006, human cases of SARS in 2003 in China, and human H7N9 cases in 2003 at its early stage. "△" means significant higher than expected, and "▽" means the reverse.

severity of clinical signs, transmission of the infectious disease, case number and the infection source (Table 1). The scores of these four aspects are then multiplied. The resulted product determines the severity of the outbreak. The higher products represent more serious outbreaks. The product in the range of 1–14, 15–29, 30–59, 60–119, 120–200 and 200–450 rate the severity of the outbreak as Grades I (mild), II (moderate), III (severe), IV (very severe), V (too severe) and VI (extremely severe), respectively.

As of April 18, 2013, the scores of the severity of clinical signs, transmission of the infectious disease, case number and the infection source were 5, 1, 3 and 3, respectively. The product of these scores was 45, which indicates that the severity of the current H7N9 influenza outbreak was at the severe level (Grade III). Its severity is lower than the SARS outbreak in China in 2003 which was of the score product 80 at its early stage [8]. In general, the severity of an infectious disease outbreak is dynamic, and it may be rated higher or lower when more characteristics of the outbreak are revealed or changed [8]. From the toll increase trend, we think that the H7N9 outbreak will become a Grade IV (very severe) outbreak at the end of this April, because the toll will exceed 100 at that time.

## 4 Possible enormous risk of the H7N9 influenza outbreak

We think that the H7N9 influenza outbreak is of enormous risk for the following reasons.

Firstly, as elucidated above, it is a Grade III (severe) and is to be a Grade IV (very severe) outbreak soon. Most of the confirmed cases developed severe symptoms, and 18.68% of them had died, indicating that the virus is of high pathogenicity to humans.

Secondly, since most of the first 91 human cases were infected with the virus from infected animals, and domestic birds infected with the virus have been identified in Anhui, Jiangsu, Zhejiang and Shanghai, indicating that the virus has spread to a wide region. In effect, the confirmed human cases and the confirmed bird infections were likely only a small portion of the reality, according to the iceberg phenomenon principle in epidemiology [9].

Thirdly, beyond that the virus can infect humans and birds, we think that the virus can also infect pigs which support circulation of both AIVs and human influenza viruses [10]. Additionally, it cannot exclude that the virus will not circulate in horse flocks after it has accumulated some mutations if it will circulate widely in the nature, as H7 subtype influenza virus had widely circulated in horses in the world for decades of the last century [11].

Fourthly, it is difficult to identify the humans, birds or pigs infected with the H7N9 virus from clinical signs, and it is extremely tough to conduct large-scale detection or surveillance to identify the virus infections in humans, birds, or pigs for a long period.

Fifthly, since the virus has spread widely, and it can infect humans, birds and pigs, and it is difficult to identify infected humans, birds and pigs, we think it is extremely tough to eliminate the virus in China, and infections of the virus in humans, birds and pigs will continue for a long period, possibly for more than ten years.

**Table 1** Scoring of four aspects of the H7N9 and SARS outbreaks at the early stage a)

| Aspects | Description | Score | H7N9 | SARS |
|---|---|---|---|---|
| Clinical signs | Mild, usually requiring treatment without hospitalization | 2 | | |
| | Severe, usually requiring hospitalization, but without severe outcomes like death, abortion or body abnormality | 3 | | |
| | Severe, <10% cases having developed severe outcomes like death, abortion or body abnormality | 4 | | √ |
| | Very severe, 10%–20% cases having developed severe outcomes like death, abortion or body abnormality | 5 | √ | |
| | Very severe, >20% cases having developed severe outcomes like death, abortion or body abnormality | 6 | | |
| Transmission | No evidences supporting person-to-person transmission | 1 | √ | |
| | Transmission through special behaviors such as sex is probable | 2 | | |
| | Transmission among frequent close contacts is probable | 3 | | |
| | Transmission among casual close contacts transmission is probable | 4 | | √ |
| | Transmission among people without close contact is probable | 5 | | |
| Case number | 1–9 | 1 | | |
| | 10–50 | 2 | | |
| | 51–100 | 3 | √ | |
| | 101–200 | 4 | | |
| | >200 | 5 | | √ |
| Infection source a) | Without links to domestic animals | 1 | | √ |
| | With a possible link to domestic animals | 2 | | |
| | With a clear evidence to support domestic animals are infection source | 3 | √ | |

a) The H7N9 outbreak data were as of 04/19/2013, while the SARS data were as of 03/09/2003.

Sixthly, due to the great populations of birds and pigs raised in China, low-level biosecurity of many live bird markets, low-level biosecurity of many small-scale poultry and swine farms in China [12], the frequent transportation of live birds and live pigs, and rapid transmission of the virus in the hosts it has adapted to, the virus will likely spread rapidly to other regions. It may be possible for the virus to spread to other countries via wild bird migration, and at least one wild bird infected with the virus has been found in Jiangsu province.

Seventhly, considering the previous circulation history of H7 subtype in some countries, the novel H7N9 virus may mutate into highly pathogenic AIV during its future probable long circulation in birds, which will be as disastrous as H5N1 highly pathogenic AIV having been circulating in many countries for years.

Eighthly, the probable long existence of the H7N9 virus in humans will provide the driving force to the virus to adapt to humans through mutations. The virus may thus obtain human-to-human transmission, and may spark a pandemic influenza thereafter. The possible pandemic should likely be very dangerous with the consideration that the virus has showed highly pathogenicity in the first 91 cases.

Some experts thought that the H7N9 outbreak was not that serious. They mainly analyzed the present status rather than its future development. Additionally, some people may think that the possible H7N9 pandemic influenza should not be that deadly, as we have just passed through the pandemic influenza caused by the novel swine H1N1 influenza virus emerging in 2009. However, influenza pandemics caused by different virus strains may be of different severity [13]. The H1N1 influenza pandemic in 2009 was mild mainly because seasonal H1N1 influenza viruses had widely circulated in humans for decades before the pandemic, which resulted in some immunity in humans against the novel pandemic H1N1 virus, especially in the elderly (so their infections were much fewer, as showed by Figure 1). In contrast, H7 subtype influenza virus has never widely circulated in humans, and thus persons of all ages are likely susceptible to the virus infection. Therefore, the future possible H7N9 pandemic will be likely more severe than the pandemic H1N1 influenza in 2009.

In light of the above considerations, we think that the H7N9 outbreak is severe, and it is likely to become very or extremely severe in the future, highlighting the emergent need of forceful scientific measures, such as building enough stockpile of H7N9 vaccine for human use in case the pandemic really occurs. The successful elimination of Nipah virus in swine population in Malaysia in 1998 and SARS coronavirus in China in 2003 has demonstrated the effect of forceful scientific measures in eliminating dangerous infectious disease outbreaks at their early stages [14,15]. Forceful scientific measures, such as massive replacement of traditional live bird markets with modern low-cost sanitary electronic live bird markets far away from residential areas [16], will be useful not only in the control of the H7N9 outbreak, but also in the control of many other infectious diseases.

## 5 Possible mild risk of the H7N9 influenza outbreak

We do not exclude completely the possibility that the H7N9 will disappear naturally, or maintain its low pathogenicity in birds and limited transmission ability in humans for a long period, without forceful scientific control measures. However, such a mild scenario of the outbreak evolution is of less possibility than the very or extremely severe scenario describe above. The main reason is that some random mutations leading to the improved adaption of the virus to humans will emerge naturally during its long existence in humans, as the virus usually evolves rapidly. These mutants will become dominant by natural selection as they are more adaptive in humans. They thus may spread in humans rapidly and can spark a deadly pandemic. Similarly, the H7N9 virus of low pathogenicity in birds is more likely to become highly pathogenic through natural selection.

Possibly only one natural force exists inhibiting the dangerous development of the H7N9 outbreak, namely the structural constraint of the virus [17]. That is to say, the mutations leading to human-to-human transmission of the virus, or leading to high pathogenicity of the virus in birds are also fatal to the virus itself. Another ideal scenario is that the virus was generated in live bird markets, and it has not spread to many poultry farms, and it will not spread to many poultry farms, and it will disappear with closure of live bird markets in related regions. We assume that some parts rather than the whole ideal scenario are possible. The future real status may be between the worst and the best scenarios given above.

*This work was supported by the Sci-tech Basic Work Project of the Ministry of Science and Technology (SQ2012FY3260033). We thank Suraj Shah in Uviversity of Illinois at Chicago for his assistance in language expression.*

1 Gao R B, Cao B, Hu Y W, et al. Human infection with a novel avian-origin influenza A (H7N9) Virus. New Engl J Med, 2013, Doi: 10.1056/NEJMoa1304459
2 Uyeki T M, Cox N J. Global concerns regarding novel influenza A (H7N9) virus infections. New Engl J Med, 2013, Doi: 10.1056/NEJMp1304661
3 Kageyama T, Fujisaki S, Takashita E, et al. Genetic analysis of novel avian A (H7N9) influenza viruses isolated from patients in China, February to April 2013. Euro Surveill, 2013, 18: 7–21
4 Jia N, Feng D, Fang L Q, et al. Case fatality of SARS in mainland China and associated risk factors. Trop Med Int Health, 2009, 14(S1): 21–27
5 National Bureau of Statistics of China. China Statistical Yearbook of 2012. Beijing: China Statistics Press, 2012
6 World Health Organization. Epidemiology of WHO-confirmed human cases of avian influenza A (H5N1) infection. Wkly Epidemiol Rec, 2006, 81: 249–257
7 Reichert T, Chowell G, Nishiura H, et al. Does glycosylation as a modifier of original antigenic sin explain the case age distribution and unusual toxicity in pandemic novel H1N1 influenza? BMC Infect Dis, 2010, 10: 5

8 Chen Y Y, Sun Y X, Chen J W, et al. Quantification of the severity of an outbreak in human infection control. Int J Infect Dis, 2010, 14: e3–5

9 Chen J M, Huang B X. Guidelines of Epidemiological Surveys of Important Animal Diseases. Beijing: Chinese Agricultural Sci-Tech Press, 2009

10 Ma W, Lager K M, Vincent A L, et al. The role of swine in the generation of novel influenza viruses. Zoonoses Public Health, 2009, 56: 326–327

11 Baigent S J, McCauley J W. Influenza type A in humans, mammals and birds: Determinants of virus virulence, host-range and interspecies transmission. Bioessays, 2003, 25: 657–671

12 China Livestock Yearbook Editorial Committee. Statistical Yearbook of China Animal Husbandry. Beijing: China Agriculture Press, 2011

13 Neumann G, Chen H, Gao G F, et al. H5N1 influenza viruses: Outbreaks and biological properties. Cell Res, 2010, 20: 51–61

14 Smith R D. Responding to global infectious disease outbreaks: Lessons from SARS on the role of risk perception communication and management. Soc Sci Med, 2006, 63: 3113–3123

15 Chen J M. A method to replace live bird market. PRC patent, 201110071333, 2012-09-19

16 Chen J M, Sun Y X. Variation in the analysis of positively selected sites using nonsynonymous/synonymous rate ratios: An example using influenza virus. PLoS One, 2011, 6: e19996

17 Chua K B. Nipah virus outbreak in Malaysia. J Clin Virol, 2003, 26: 265–275

**Open Access** This article is distributed under the terms of the Creative Commons Attribution License which permits any use, distribution, and reproduction in any medium, provided the original author(s) and source are credited.
